# Sex differences in emotional perception: Evidence from population of Tuvans (Southern Siberia)

**DOI:** 10.3389/fpsyg.2022.924486

**Published:** 2022-09-13

**Authors:** A. A. Mezentseva, V. V. Rostovtseva, K. I. Ananyeva, A. A. Demidov, M. L. Butovskaya

**Affiliations:** ^1^Institute of Ethnology and Anthropology of the Russian Academy of Sciences, Moscow, Russia; ^2^Institute of Psychology of the Russian Academy of Sciences, Moscow, Russia; ^3^Moscow Institute of Psychoanalysis, Moscow, Russia; ^4^National Research University Higher School of Economics, Moscow, Russia; ^5^Russian State University for the Humanities, Moscow, Russia

**Keywords:** emotion recognition, facial expressions, sex differences, anger, Tuvans

## Abstract

Prior studies have reported that women outperform men in nonverbal communication, including the recognition of emotions through static facial expressions. In this experimental study, we investigated sex differences in the estimation of states of happiness, anger, fear, and disgust through static photographs using a two-culture approach. This study was conducted among the Tuvans and Mongolian people from Southern Siberia. The respondents were presented with a set of photographs of men and women of European and Tuvan origin and were asked to interpret each of them. They were asked: “What does the person in the photo feel?” We found that the Tuvans easily identified happiness and anger; however, the level of accuracy of fear and disgust recognition was low. No sex differences in the recognition of happiness, disgust, and fear were observed. However, anger recognition was significantly moderated by the perceiver’s sex and the origin of the model. Compared to Tuvan men, Tuvan women were significantly less accurate in identifying anger in male Tuvans. Furthermore, the age effect was found in recognition of fear: older Tuvans were more accurate while recognizing the fearful faces of Tuvan, but not the European models.

## Introduction

The theoretical basis for studies of human emotions and related facial expressions dates back to Darwin’s book, *The Expression of the Emotions in Man and Animals* (Darwin, 1872). Earlier, he discussed the evolutionary foundations of emotions and their expressions in his book *The Descent of Man, and Selection in Relation to Sex* (Darwin, 1871). So far, the questions of the universality of expression and recognition of basic human emotions remain one of the most popular topics for evolutionary psychologists and human ethologists.

Previous studies that examined various populations found that women were more accurate than men in judging basic emotional states from static facial images by means of forced choice (see the meta-analyses of Thompson and Voyer, 2014). The effect sizes in this study were approximately Cohen’s *d* = 0.4. Same conclusions were drawn from the most recent study of facial emotion recognition through videos, where the results demonstrated robust sex differences favoring women (Cohen’s *d* = 0.54, Wingenbach et al., 2018). Sex differences, where women outperform men in non-verbal communication abilities, have been found across different age cohorts, including infants, children, and adults, with the largest age effects on anger, fear, sadness, and no effect on disgust (see meta-analysis: Hayes et al., 2020). Interestingly, the largest effect size for sex differences has been found in infants, favoring girls in their ability to recognize emotional facial expressions according to a meta-analysis (McClure, 2000). However, the forced-choice approach has apparent limitations, as it suggests solutions by offering a ready-made option (Lorette, 2021). Recently, an increasing number of studies have adopted a different approach in which observers are offered to freely label emotional facial expressions (Gendron et al., 2018). In this study, we used this method in particular. To the best of our knowledge, this is the first study that tests sex differences in emotional recognition using free-labelling approach.

Despite an abundance of studies, the factors that moderate human emotion recognition remain poorly understood to this date. Recently, it has been pointed out that the type of emotion is one of the factors that moderate recognition of human emotions. For instance, Thompson and Voyer’s (2014) meta-analytic research showed that sex differences in recognition of basic emotions from stimuli of various modalities (visual, auditory, and mixed) reach the largest effect sizes when identifying angry, fearful, and disgusted states (around *d* = 0.2), with anger having the largest effect compared to other emotions combined. However, Wingenbach et al. (2018) reported no significant interaction between observers’ sex and the type of recognized emotion. From an evolutionary perspective, the foundations of female hypersensitivity to negative emotions may be rooted in the psychological differences between males and females related to maternal behavior. Such differences could also occur partly because of women’s inherent role as a primary caretaker (“primary caretaker hypothesis,” Babchuk et al., 1985), suggesting that women are capable of recognizing infant emotional cues more accurately. It concerns the ability to recognize potential threats to the infant’s survival by detecting and analyzing negative emotions (see also “fitness threat hypothesis,” Hampson et al., 2006). Numerous studies have shown the impact of the model’s sex on the perception of emotions. Some of them demonstrated that women perceive emotions more accurately in male models, whereas the model’s sex did not affect recognition of emotions in men (Rahman et al., 2004). Other studies reported that the sex of model influenced neither the female nor the male ability to recognize emotions (Thayer and Johnsen, 2000). Older adults were generally reported to have poorer recognition of facial expressions (see meta-analyses: *N* = 10,526; Hayes et al., 2020).

The current study investigated facial emotion recognition among non-WEIRD (western, educated, industrialized, rich, and democratic) populations of traditional Mongolian origin nomadic pastoralists, Tuvans, who are settled in one of the most inaccessible regions of Russia (and most recently joined) in Southern Siberia, and thereby have limited contact with the Western culture. Special interest in studying the Tuvan population concerns their social environment as until recently, they remained a highly monoethnic, patriarchal society, predominantly speaking the Tuvan language and following cultural traditions in daily life (e.g., sex-based division of labor; Lindquist, 2008). Relevant communication studies in Mongolian cultures suggest that Tuvans are more predisposed to expressing emotions through gestures than through facial expressions (Sereedar, 2011). In this study, we suggested that such a specific cultural environment could moderate their ability to recognize emotional states through facial expressions. Thus, this study investigated whether facial emotion recognition differed as a function of participant’s sex, stimuli sex and ethnicity origin of facial stimuli (European vs. Mongolian). We predicted that women would be better at recognizing anger, fear, and disgust than men, whereas no sex differences would be observed in the recognition of happiness. While exploratory in nature, we also predicted that Tuvans would evaluate facial emotion expressions differently between Caucasian and Mongolian faces.

## Materials and methods

### Data collection and participants

The participants of our study were Tuvans, a nomadic pastoralist population of Mongolian origin from Southern Siberia (Hooper, 2020), who speak the Siberian Tuvan language, Tuvan. Today, most of the Tuvans live in the territory of the Tuva Republic (approximately 260,000 people), one of the most culturally isolated regions of Russia bordering Mongolia in the south. Traditionally, the Tuvan society was organized as patrilineal clans, which formed the primary identity of people and determined their legal rights (Lindquist, 2008). They consider themselves Buddhists (Lamaism); however, a fraction of the Tuvans define themselves as shamanists. It is noteworthy that the Tuva Republic joined Russia (USSR) only in 1944. This population speaks in the Tuvan language for everyday communication (most of them know only a few Russian words) and remains predominantly monoethnic to date. We conducted our research in a rural settlement, Erzin, located in a natural conservation area, Ubsunur Hollow. To date, Erzin residents have had limited contact with the European population and limited exposure to the Western culture. We collected data from 67 individuals aged 18–45 years (35 men and 32 women, mean age 29.5 years, SD = 9.29) in August 2021. According to Tuvans, the age group considered for this study had similar cultural norms and social status.

### Stimuli

This study used color photographs of four Caucasians (two men and two women) and four Tuvan models (two men and two women) exhibiting emotional facial expressions (happiness, anger, fear, and disgust) as stimuli. When selecting models, we considered the age range of the research cohort (18–45 years); consequently, we selected photos of middle-aged men and women of the similar age range. Images of Caucasians were obtained from the FACES database (Ebner et al., 2010). Due to their rarity, stimuli of Siberian Mongolian origin, Tuvans, were created by us particularly for this project. For this purpose, four Tuvan models (actors recruited on a voluntary basis from the capital of Tuva) were invited to display the target facial expressions in front of the camera. To ensure that the stimuli data were reliable, we conducted a pilot forced-choice research using an online form, in which 120 Moscow residents, aged 18–45 years, participated. According to the results, stimulus images were considered sufficiently reliable to be used in the study (for more details, see Supplementary Table 1). The final set of stimuli contained 32 cards (eight faces × four facial expressions). A neutral facial expression of the same model was displayed on each card along with one facial emotional expression each.

### Design and procedure: free-labelling facial configuration

Each participant was presented with 32 stimulus cards: (European: 2 men + 2 women + Tuvan: 2 men + 2 women) × four emotional expressions: *anger*, *disgust*, *fear*, and *happiness*. Stimulus cards were displayed randomly on a laptop screen with no time limit for viewing. During the evaluation of each emotion, the experimenter asked the respondent to describe what was shown in the image. The respondent was asked: “What does the person in the image feel?” Next, the participants’ answers were noted by the experimenter’s assistant (Tuvan representative). Before this, each participant was asked whether they knew the model in person. The assistant provided the translation (Russian-Tuvan and vice versa) and necessary explanations when required. The last response was fixed as the final answer, which was accepted after clarifying all questions of the interviewee (for more details, see Supplementary Table 2).

### Coding

The participants’ responses were coded into a binary variable: 1 (able to guess) was assigned to the cases when the intended emotional facial expression from the stimulus card corresponded to an emotional label (or synonyms) stated by a participant (for synonyms, see Supplementary Table 2). In cases where there was a mismatch, 0 (not able to guess) was assigned. To test the intercoder reliability, we engaged two observers who recorded answers from 10 randomly selected participants’ interviews (five Tuvan men and five Tuvan women) independently. The inter-observer agreement was found to be almost perfect (Cohen’s kappa, *κ* = 0.92, *p* < 0.0001). Finally, a rough clarifying approach was applied to the resulting answers, and it was believed that an expert understood an emotional facial expression only if he/she correctly and accurately recognized the corresponding emotion on two similar stimuli (models of the same sex and origin). Same sex models and their origin were factors necessary to remove possible effects related to the individual facial features of the models. Same sex models and their origin were factors necessary to remove possible effects related to the individual facial features of the models that could influence emotion recognition.

### Statistical analysis

We used a binomial test to assess the consistency of the Tuvans when they labelled four given emotional expressions (anger, happiness, disgust, and fear), with 0.16 set as an expected chance level consistency [based on the number of available and known basic emotional states (six)]. The binomial model included participants’ answers received for each emotional category. The ability to correctly guess a facial expression was set as the binary response variable, which was calculated by averaging the results from similar stimuli (models of the same sex and origin). Only in the cases where the perceiver was able to correctly guess the facial expression from both similar stimuli, the response variable was set to 1 (able to guess); in other cases, it was set to 0 (not able to guess).

To assess the impact of the models’ sex and origin, and that of the perceivers on the ability to correctly distinguish between emotional facial expressions, we used generalized estimating equations (GEE), a binary logistic model with repeated measures, conducted for each emotional expression separately. Here, we used the encoding technique described above. There were four measurements per subject (for each emotional expression). The statistical model assessed both the main effects and the interactions between the independent variables. We conducted a detailed analysis, where binary variables (sex and ability to guess emotions) were matched using the chi-squared criterion to focus on the obtained significant effects.

## Results

According to the binomial test, the easiest emotion to identify for the participants was happiness [95% of participants (*p* < 0.000) correctly labelled the smiling configuration] followed by anger [66% of Tuvans (*p* < 0.000) correctly labelled the crowing facial configuration]. However, Tuvans tended to show a low accuracy rate for identifying fear (25% identified, *p* < 0.000) and disgust (22% identified, *p* < 0.006). All results were statistically significant, with an expected chance level consistency of 0.16.

**Figure 1:**
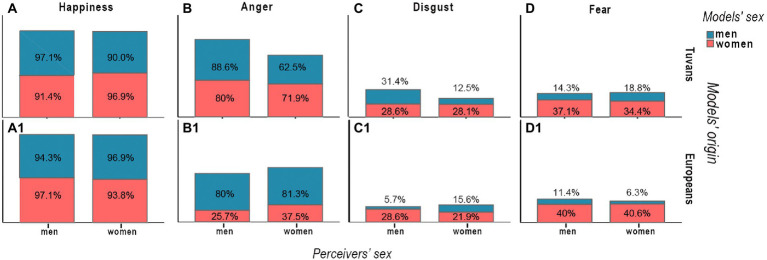
Relative frequencies of right guesses, where general number of perceivers of the same sex estimating models of the same sex and origin are taken as 100%.

The frequency of correct guesses of emotional facial expressions is shown in Figure 1. It was slightly easier for the Tuvans to identify happiness, anger, and disgust through facial expressions of same-sex Tuvans, implying that Tuvan men were slightly better at recognizing these emotions on the faces of Tuvan men and women on the faces of Tuvan women (Figures 1A–C). Fear was better recognized on female faces by both male and female perceivers, independent of the model’s origin (Figures 1D,D1). Although there were almost no sex differences in the perception of happiness through faces of European models (Figure 1A1), both male and female Tuvans better recognized anger on male, but not female, European faces (Figure 1B1).

To test the significance of these results, we applied binary logistic models with repeated measurements to assess the impacts of the models’ and perceivers’ sex, model origin, and perceiver’s age.

According to the results of the statistical analysis, the ability to correctly guess disgust and happiness was affected neither by the model’s sex and origin nor by the sex and age of the perceivers (for detailed statistics, see Supplementary Tables 3, 4). Similarly, the recognition of anger and fear was significantly affected by several independent factors. The sex and origin of the models as well as the sex of the perceiver had a significant impact on correctly identifying anger (Table 1), whereas recognition of fear was affected by the model’s origin and the perceiver’s age (Table 2).

**Table 1:** Effects of models’ sex and origin, and sex and age of perceivers on ability to guess anger.

Dependent var.: Guess anger			
Predictors	*B*	Wald chi-squared	*p* (sig.)
Models’ sex	−0.501	0.378	0.539
Models’ origin	−1.431	2.681	0.102
Perceivers’ sex	1.181	0.822	0.364
Perceivers’ age	0.014	0.170	0.680
**Models’ sex × Models’ origin**	**2.142**	**18.533**	**<0.001**
Models’ sex × Perceivers’ sex	0.749	1.708	0.191
Models’ sex × Perceivers’ age	0.007	0.065	0.799
**Models’ origin × Perceivers’ sex**	**−1.274**	**4.946**	**0.026**
Models’ origin × Perceivers’ age	0.004	0.016	0.898
Perceivers’ sex × Perceivers’ age	−0.020	0.253	0.615

GEE, binary logistic model with repeated measures. The amount of measurements taken per subjects is 4. The amount of subjects is 67. The significant predictors are shown in bold.

**Table 2:** Effects of models’ sex and origin, and sex and age of perceivers on ability to guess fear.

Dependent var.: Guess fear			
Predictors	*B*	Wald chi-squared	*p* (sig.)
Models’ sex	0.207	0.022	0.883
**Models’ origin**	**1.742**	**4.378**	**0.036**
Perceivers’ sex	1.360	0.698	0.403
**Perceivers’ age**	**0.096**	**6.943**	**0.008**
Models’ sex × Models’ origin	−0.934	2.351	0.125
Models’ sex × Perceivers’ sex	0.015	0.001	0.981
Models’ sex × Perceivers’ age	−0.042	1.257	0.262
Models’ origin × Perceivers’ sex	0.146	0.085	0.770
**Models’ origin × Perceivers’ age**	**−0.053**	**4.220**	**0.040**
Perceivers’ sex × Perceivers’ age	−0.047	0.900	0.343

GEE, binary logistic model with repeated measures. The amount of measurements taken per subjects is 4. The amount of subjects is 67. The significant predictors are shown in bold.

We conducted additional analyses to address the independent factors that affected recognition of anger and fear while focusing on the obtained significant effects. The analyses revealed that all Tuvan women, compared to Tuvan men, were less frequently correct in guessing anger through facial expressions in male Tuvan models (chi-squared test for independence: *N* = 67, *χ*^2^ = 6.2, *df* = 1, *p* = 0.02). However, all other effects of sex on the perception of anger stimuli were insignificant, and there were no sex differences in recognition of anger in female models of Tuvan origin (*N* = 67, *χ*^2^ = 0.6, *df* = 1, *p* = 0.568), as well as in female (*N* = 67, *χ*^2^ = 0.998, *df* = 1, *p* = 0.430) and male (*N* = 67, *χ*^2^ = 0.17, *df* = 1, *p* = 1.0) models of European origin.

**Figure 2:**
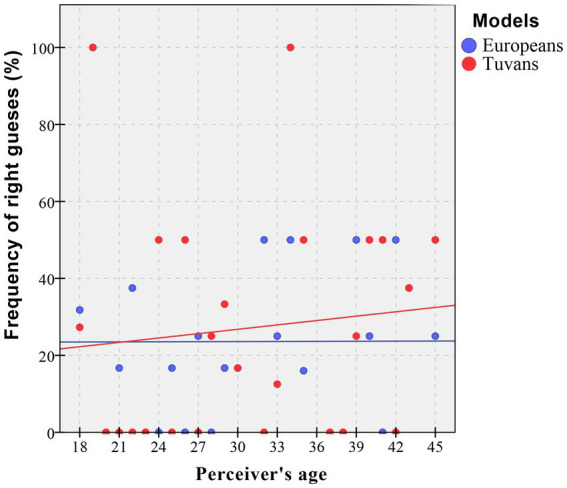
Impact of perceivers’ age on fear recognition in models of European and Tuvan origin.

According to the binary logistic model, the recognition of fear was affected by the model’s origin and age (Table 2). This effect was visualized in Figure 2. Relying on the visualization, the older the perceivers, the more accurate they were in recognizing fear in Tuvan models of both sexes.

## Discussion

The study was conducted in an infrastructurally and geographically isolated settlement in Southern Siberia, among Tuvans. Until now, it has remained a strongly patriarchal culture. Here, we studied whether the accuracy of estimations of emotional statuses differed between sexes and other moderating factors.

Our data revealed that the easiest emotions to identify for the Tuvans (both men and women) were happiness and anger; however, they tended to show low accuracy rates for fear and disgust. Similar results have been reported in many other East Asian populations before (Matsumoto and Ekman, 1989). It can be argued that East Asians, compared to representatives of Western culture, process faces differently; they pay more attention to the eye region. Thus, the lack of focus on the mouth may contribute to the systematic misclassification of facial expressions: disgust as anger and fear as surprise (Tan et al., 2015).

Contrary to what was expected, we found no effect of the ethnic origin of stimuli (European vs. Mongolian) on emotional recognition. The Tuvans did not evaluate facial emotional expressions between European and Mongolian faces differently (there was no lack of recognition of emotions on European faces). Similar conclusions were drawn in one of the previous studies, where no differences in the perception of facial features of Buryats (Mongolian people of Southern Siberia) were found between Caucasian and Mongolian raters (Russians and Buryats; Rostovtseva et al., 2022). We believe that these results can be explained by the impact of global international media (Internet, television, and cinema), which provides an opportunity for audiences from different parts of the world to become acquainted with different types of human appearances.

We found an age effect in fear recognition: older Tuvans were better at recognizing fearful faces of Tuvan, but not European models. Despite the fact that previous studies reported that older adults were typically worse at fear recognition due to general aging processes (reviewing Isaacowitz and Stanley, 2011), in our case, the upper margin of participants’ age was only 45 years. Thus, we suppose that older individuals are more experienced in communicating with representatives of their own population and, thus, may be more sensitive to some aspects of emotional recognition.

Unlike most previous studies, which used a forced-choice format, we found no sex differences in the recognition of happiness, disgust, and fear. Moreover, independent factor analyses revealed that all Tuvan women were less successful in recognizing anger than Tuvan men, in the case of Tuvans’ male models. However, to our knowledge, a pattern in which women were inferior to men in the perception of negative, threat-signaling emotional status has not been discovered. One possible explanation for this is women’s poor communication abilities, a result of cultural conditioning. Even today, Mongolian cultural traditions encourage gender division of labor, where women’s occupation and social environment are limited to the household (Anzhiganova and Ak-Lama, 2016; Natsak, 2022), and prescribe limited contact with strangers of opposite sexes. Nevertheless, the reasons for Tuvan women’s particular insensitivity to men’s angry facial expressions remain poorly understood and require further research.

Our study had certain limitations. The significance level of the sex-based difference in anger recognition did not survive the Bonferroni correction for multiple comparisons. One possible reason for this could be the small sample size, which, in turn, was due to the nature of the small-scale population that was studied. Another limitation was that we used posed emotional expressions as stimuli rather than genuine ones. However, this is a general limitation of such studies.

## Data availability statement

The datasets presented in this study are available upon reasonable request from the corresponding author.

## Ethics statement

The study was approved by the Ethical Committee of the Institute of Ethnology and Anthropology of the Russian Academy of Sciences. All subjects and models signed informed consents prior to the experiment.

## Author contributions

AM, MB, and VR contributed to conception and design of the study and drafted the manuscript. AM, VR, AD, and KA collected the data. AM processed the raw data and organized the database. VR and AM performed the statistical analysis. All authors contributed to the article and approved the submitted version.

## Funding

The article was prepared in the framework of a research grant funded by the Ministry of Science and Higher Education of the Russian Federation (grant ID: 075-15-2020-910; AM, VR, MB). The data collection was supported by a grant of Russian Foundation for Basic Research No. 20-313-70005 (KA).

## Conflict of interest

The authors declare that the research was conducted in the absence of any commercial or financial relationships that could be construed as a potential conflict of interest.

## Publisher’s note

All claims expressed in this article are solely those of the authors and do not necessarily represent those of their affiliated organizations, or those of the publisher, the editors and the reviewers. Any product that may be evaluated in this article, or claim that may be made by its manufacturer, is not guaranteed or endorsed by the publisher.
