# Supplementary material for: Sex differences in emotional perception: Evidence from population of Tuvans (Southern Siberia)
Source: Front Psychol. 2022 Sep 13;13:924486. doi: 10.3389/fpsyg.2022.924486 (PMC9513426; doi:10.3389/fpsyg.2022.924486)
Supplement: Supplementary file 1 [file Data_Sheet_1.PDF]

Supplementary materials

Table 1

Mean Percentage Endorsement of Emotion Label by Face in Forced Choice Task										
Face ID	Population origin	Gender	Set	Emotion Portrayed	Emotion Endorsed					
					Anger	Disguist	Fear	Happiness	Sadness	Suprise
SMY	Caucasian	Male	Faces	Happiness	1.12%	4.08%	0.0%	**92.86%**	2.25%	0.0%
SMY	Caucasian	Male	Faces	Disguist	0.0%	**98.88%**	0.0%	0.0%	0.0%	1.12%
SMY	Caucasian	Male	Faces	Fear	1.12%	1.12%	**46.5%**	0.0%	0.0%	51.18%
SMY	Caucasian	Male	Faces	Anger	**97.75%**	1.12%	1.12%	0.0%	0.0%	0.0%
SME	Caucasian	Male	Faces	Happiness	0.0%	0.0%	0.0%	**100.0%**	0.0%	0.0%
SME	Caucasian	Male	Faces	Disguist	0.0%	**97.75%**	2.25%	0.0%	0.0%	0.0%
SME	Caucasian	Male	Faces	Fear	0.0%	1.12%	**82.02%**	0.0%	0.0%	16.85%
SME	Caucasian	Male	Faces	Anger	**82.02%**	11.24%	0.0%	0.0%	4.49%	2.25%
SWY	Caucasian	Female	Faces	Happiness	0.0%	0.0%	0.0%	**100.0%**	0.0%	0.0%
SWY	Caucasian	Female	Faces	Disguist	0.0%	**89.89%**	0.0%	0.0%	8.99%	1.12%
SWY	Caucasian	Female	Faces	Fear	1.12%	1.12%	**65.17%**	0.0%	0.0%	32.58%
SWY	Caucasian	Female	Faces	Anger	**100.0%**	0.0%	0.0%	0.0%	0.0%	0.0%
SWE	Caucasian	Female	Faces	Happiness	0.0%	0.0%	0.0%	**100.0%**	0.0%	0.0%
SWE	Caucasian	Female	Faces	Disguist	0.0%	**96.63%**	0.0%	0.0%	3.37%	0.0%
SWE	Caucasian	Female	Faces	Fear	0.0%	3.37%	**77.53%**	0.0%	2.25%	16.85%
SWE	Caucasian	Female	Faces	Anger	**47.19%**	8.99%	2.25%	2.25%	37.08%	2.25%
TMY	Mongolian	Male	our data	Happiness	0.0%	0.0%	0.0%	**100.0%**	0.0%	0.0%
TMY	Mongolian	Male	our data	Disguist	4.49%	**82.02%**	0.0%	0.0%	3.37%	10.11%
TMY	Mongolian	Male	our data	Fear	1.12%	2.25%	**34.83%**	0.0%	0.0%	59.55%
TMY	Mongolian	Male	our data	Anger	**86.52%**	11.24%	0.0%	1.12%	1.12%	0.0%
TME	Mongolian	Male	our data	Happiness	1.12%	1.12%	0.0%	**96.63%**	1.12%	0.0%
TME	Mongolian	Male	our data	Disguist	0.0%	**94.38%**	0.0%	0.0%	4.49%	1.12%
TME	Mongolian	Male	our data	Fear	0.0%	0.0%	100.0%	0.0%	0.0%	0.0%
TME	Mongolian	Male	our data	Anger	**88.76%**	3.37%	0.0%	0.0%	7.78%	0.0%
TWE	Mongolian	Female	our data	Happiness	0.0%	0.0%	0.0%	**100.0%**	0.0%	0.0%
TWE	Mongolian	Female	our data	Disguist	17.98%	**77.53%**	0.0%	0.0%	2.25%	2.25%
TWE	Mongolian	Female	our data	Fear	1.12%	0.0%	**98.88%**	0.0%	0.0%	0.0%
TWE	Mongolian	Female	our data	Anger	**47.19%**	50.56%	0.0%	0.0%	2.25%	0.0%
TWY	Mongolian	Female	our data	Happiness	0.0%	0.0%	0.0%	**100.0%**	0.0%	0.0%
TWY	Mongolian	Female	our data	Disguist	0.0%	**93.26%**	0.0%	0.0%	6.74%	0.0%
TWY	Mongolian	Female	our data	Fear	0.0%	5.62%	**75.28%**	0.0%	8.99%	10.11%
TWY	Mongolian	Female	our data	Anger	**83.15%**	5.62%	0.0%	0.0%	11.24%	0.0%

Note. Percentages represent the percent of participant that selected a given label as the best descriptor for a given face. Cells that are bolded reflect the percentage of participants who indicated that the face most clearly portrayed the intended emotion. Data were collected from 120 participants, 120 Moscow residents, aged 18-45. Each individual rated all 32 faces in a forced-choice task. Images of the original stimuli used can be provided upon request.

Table 2

**Descriptions directly corresponding to intended emotions**	
Happiness	happy, cheerful, joy, good mood, relief
Disguit	disguist, queasiness, squeamishness, stink
Fear	fear, fright, horror
Anger	anger, the desire to scare and show strength, aggression, furiousness, irascibility
**Other descriptions, which were not corresponding to intendent discreet emotions directly**	
Surprise	shock, surprise
Sadness	sadness, resentment, frustration
Irritation	shyness, embarrassment
Pain	headache
Discontent	frowns, dissatisfied with something, disliked something, feeling unwell, negative mood
Incomprehension	lack of understending: the model is just sitting/looking/thinking/photographing/ grimacing/ drunk

Note. Only in those cases when perceiver was able to correctly guess the facial expression (to provide descriptions, which were directly corresponding to the intended emotion) the response variable was set to 1 (able to guess), in other cases it was set to 0 (not able to guess).
